# Risk Factors for *Plasmodium falciparum* Gametocyte Positivity in a Longitudinal Cohort

**DOI:** 10.1371/journal.pone.0123102

**Published:** 2015-04-01

**Authors:** Laura Grange, Cheikh Loucoubar, Olivier Telle, Adama Tall, Joseph Faye, Cheikh Sokhna, Jean-François Trape, Anavaj Sakuntabhai, Jean-François Bureau, Richard Paul

**Affiliations:** 1 Institut Pasteur, Unité de la Génétique Fonctionnelle des Maladies Infectieuses, 28 rue du Docteur Roux, F-75724 Paris cedex 15, France; 2 Centre National de la Recherche Scientifique, URA3012, F-75015 Paris, France; 3 Institut Pasteur de Dakar, Unité d’Epidémiologie des Maladies Infectieuses (UR 172), Dakar, Senegal; 4 Institut de Recherche pour le Développement, Dakar, Unité de Pathogénie Afro-Tropicale (UMR 198), Senegal; Shoklo Malaria Research Unit Mahidol University and University of Oxford, THAILAND

## Abstract

Malaria transmission intensity is highly heterogeneous even at a very small scale. Implementing targeted intervention in malaria transmission hotspots offers the potential to reduce the burden of disease both locally and in adjacent areas. Transmission of malaria parasites from man to mosquito requires the production of gametocyte stage parasites. Cluster analysis of a 19-year long cohort study for gametocyte carriage revealed spatially defined gametocyte hotspots that occurred during the time when chloroquine was the drug used for clinical case treatment. In addition to known risk factors for gametocyte carriage, notably young age (<15 years old) and associated with a clinical episode, blood groups B and O increased risk compared to groups A and AB. A hotspot of clinical *P*. *falciparum* clinical episodes that overlapped the gametocyte hotspots was also identified. Gametocyte positivity was found to be increased in individuals who had been treated with chloroquine, as opposed to other drug treatment regimens, for a clinical *P*. *falciparum* episode up to 30 days previously. It seems likely the hotspots were generated by a vicious circle of ineffective treatment of clinical cases and concomitant gametocyte production in a sub-population characterized by an increased prevalence of all the identified risk factors. While rapid access to treatment with an effective anti-malarial can reduce the duration of gametocyte carriage and onward parasite transmission, localised hotspots represent a challenge to malaria control and eventual eradication.

## Introduction

Global efforts to control and eliminate *Plasmodium falciparum*, the etiological agent of malignant tertian malaria, over the last decade have resulted in a 40–50% decrease in malaria mortality rates. In 2012, there were an estimated 207 million cases of malaria and 627 000 malaria deaths [1]. In addition to improved diagnostics, the decrease in the malaria burden has coincided with increased deployment of insecticide treated bednets (ITNs) and use of Artemisinin-based combination therapy (ACT), both of which likely interfere with transmission of the parasite. ITNs not only protect individuals from receiving an infectious mosquito bite, but will also prevent infected individuals from transmitting the parasite to uninfected mosquitoes. ACTs are not only effective against the asexual parasite stages responsible for disease, but may also impact upon the stages transmissible to mosquitoes [2]. However, the emergence of drug and insecticide resistance [3, 4] is cause for concern and, in the absence of any effective vaccine, additional strategies to reduce malaria transmission are needed.

It has long been recognised that there exists very fine scale spatial heterogeneity in the epidemiology of malaria, even at the scale of the within-village community [5–8]. Major components determining this spatial heterogeneity include household proximity to mosquito breeding/oviposition sites, and very local, household level environmental factors influencing adult mosquito distribution, biting behaviour and longevity [9, 10]. Spatial heterogeneity in malaria epidemiology has been theoretically shown to increase the effective R_0_ [11–13] and there is concerted interest in the development of interventions to target transmission hotspots and/or individuals with increased risk of infection [14].

Transmission of malaria parasites from man to mosquito depends on the production of gametocyte sexual parasite stages in the human host that are subsequently taken up by a mosquito during a bloodmeal [15]. For *P*. *falciparum*, sexual stage differentiation (gametocytogenesis) from asexual parasites occurs in the blood of the human host. Although all parasite infections produce gametocytes at some point during the infection, their frequency and density varies. In cases of clinical malaria, gametocyte carriage has been associated with a worsening blood environment for the parasite (e.g. fever responses, anaemia, and the presence of reticulocytes) [16–18]. However, under intense transmission, clinical immunity develops during childhood after many infections [19, 20], whereby the individual can tolerate non-negligible parasite densities without showing symptoms. Such asymptomatic infections can also generate gametocytes and infect mosquitoes [15, 21–23].

We have previously shown that there is a significant human genetic contribution to gametocyte carriage in asymptomatic infections [24]. The sickle cell mutation, HbS, which is protective against severe disease [25] was associated with increased gametocyte prevalence but its contribution was small. Others have shown that individuals with beta-globin variants who are infected with *P*. *falciparum* are more infectious to mosquitoes [26]. These findings suggest that there may exist superspreaders—individuals who contribute significantly to the transmission of malaria at a local scale [27]. Here we extend our previous work to identify factors impacting upon gametocyte carriage in a 19-year longitudinal malaria dataset of a village cohort. We then perform a spatio-temporal cluster analysis to identify potential gametocyte hotspots and examine the differential impact of the observed risk factors on the spatial and temporal bounds identified in the cluster analysis.

## Material and Methods

### Participants

Between 1990 and 2008, a longitudinal study involving the inhabitants of the village of Dielmo, Senegal, was carried out to identify all episodes of fever. The study design included daily medical surveillance with systematic blood testing of individuals with fever and examination of 200 oil-immersion fields on a thick blood film for malaria parasites (about 0.5 μL of blood). In addition, monthly systematic blood slides were taken from participating individuals to assess the prevalence rate of asymptomatic infections. The village is situated in a Sudan-savannah region of central Senegal, on the marshy bank of a small permanent stream, where anopheline mosquitoes breed all year round [28, 29]. Malaria transmission is intense and perennial, with a mean 258 infected bites per person per year during 1990–2006 [30, 31]. As previously described [32], the family structure (pedigree) was available after a demographic census performed for every volunteer at his adhesion in the project. A verbal interview of mothers or key representatives of the household was used to obtain information on genetic relationships between studied individuals, their children, their parents, and to identify genetic links among the population. The total pedigree comprised 828 individuals, including absent or dead relatives, composed of ten independent families that can be sub-divided into 206 nuclear families (father—mother couples with at least one child) with an average of 3.6 children each. Genetically related nuclear families occur because of multiple marriages and marriages among related individuals. Previous typing with microsatellites has enabled the construction of a pedigree based on Identity-by-Descent using MERLIN [33, 34].

### Ethical approval

The malaria research program conducted in Dielmo village in Senegal has been ongoing since 1990 as described elsewhere [28, 29]. The project was approved by the Ministry of Health of Senegal and the assembled village population. Approval is renewed on a yearly basis. The project protocol and objectives were carefully explained to the assembled village population. Written informed consent was obtained from all participants in our study over the age of 15 years or the guardians of children younger than 15 years. In all cases, such consent was obtained in the presence of the school director, an independent witness. Written consent was recorded on a voluntary consent form written in both French and Wolof, the local language. This methodology was approved by the National Ethics Committee of Senegal, which carries out audits regularly along with *ad hoc* committees of the Ministry of Health, the Pasteur Institute (Dakar, Senegal) and the Institut de Recherche pour le Développement (Marseille, France).

### Procedures

Each individual was given a unique identification code and details of family ties, occupation, and precise place of residence were recorded on detailed maps of each household with the location of each bedroom. All households were visited daily, absenteeism recorded, and the presence of fever or other symptoms assessed. We systematically recorded body temperature at home three times a week (every second day) in children younger than 5 years, and in older children and adults in cases of suspected fever or fever-related symptoms. In cases of fever or other symptoms, blood testing was done at the dispensary by finger prick, and we provided detailed medical examination and specific treatment. Parasitologically confirmed clinical malaria episodes were treated according to national guidelines. From 1990 to 2008, four different drug regimens were implemented: Quinine from 1990 to 1994, Chloroquine from 1995 to 2003, Fansidar (sulfadoxine-pyrimethamine) from 2004 to mid-2006 and Artemisinin-based combination therapy (ACT; Amodiaquine- sulfadoxine-pyrimethamine) from mid-2006 to 2008.

Parasite positivity was established as follows. Thick blood films were prepared and stained by 3% Giemsa stain. Blood films were examined under an oil immersion objective at x1000 magnification by the trained laboratory technicians and 200 thick film fields were examined to count the number of asexual and gametocyte parasite stages. Asexual parasite densities (per μL) were calculated by establishing the ratio of parasites to white blood cells and then multiplying the parasite count by 8,000, the average white blood cell count per μL of blood.

The outcome variable of interest was whether a bloodslide taken at clinical presentation or collected within the framework of the systematic follow up contained gametocytes, regardless of the disease status or time since last drug treatment. Thus, we assess the prevalence of gametocytes irrespective of the cause. The duration of gametocyte carriage for a single infection in endemic settings can last for at least up to 30 days [35]. To increase the probability that only independent episodes of gametocyte production from the same individual are included, we first considered gametocyte positivity per trimester. We thus first aggregated the data per trimester per individual and recorded whether they were gametocyte positive at any time during the trimester. Some explanatory variables were time-dependent and were therefore evaluated for each trimester. These included current age, occurrence of infection with other *Plasmodium* spp. (*Plasmodium ovale* and *Plasmodium malariae*), occurrence of *P*. *falciparum* clinical episodes and maximum parasite density. Other variables were individual-dependent including sex, sickle cell trait and the alpha-globin deletion as previously characterized [24]. ABO blood group was characterized by the Beth-Vincent test and confirmed by the Simonin-Michon test. An exhaustive list of variables with univariate analysis results is available in S1 Table.

In a second analysis, to address the impact of time since previous drug treatment on gametocyte positivity, the data were not aggregated into trimester.

### Statistical analyses

#### Risk factor analysis

To evaluate the effects of the explanatory variables on the risk of gametocyte carriage during a trimester, we used Generalized Linear Mixed Models (GLMM) using Genstat ver. 15 (VSN Ltd) to account for the non-independence of individuals because of family relationships and for repeated measures from the same individual [36]. Correlated individual effects due to familial relationships were taken into account by using the pedigree-based genetic relatedness matrix that contains the genetic covariance among all pairs of individuals in the study cohort and is calculated using the pedigree information [37]. Genetic covariance and repeated measures from the same individual were modelled as random effects. Occurrence or not of gametocytes was modelled as a binary outcome. Initially explanatory variables were tested individually in univariate analyses and Wald statistics, which approximate to a χ^2^ distribution, were established. All variables having a *P* value ≤0.25 were tested in a multivariate analysis (S1 Table). Model simplification was carried out by fitting a GLMM with all the variables (P<0.25) and removing the variables one by one on the basis of the change in. the Akaike Information Criterion (AIC). I.e. at each step, each of the variables was removed and replaced and the change in the AIC noted. The variable whose removal lead to the largest decrease in AIC at each step was then removed and this procedure was repeated until there remained only statistically significant variables in the model (S2 Table).

For confirmation of the hotspots identified in SaTScan (see below)[38], a GLMM model was fitted with individuals being classified by a factor “in” or “out” of the hotspot. To assess the differential effect of the global risk factors on gametocyte positivity in infected individuals “in” or “out” of the hotspot during the time span covered by the hotspots, separate analyses were conducted for each group of individuals.

#### Spatial cluster analysis

**Fig 1:**
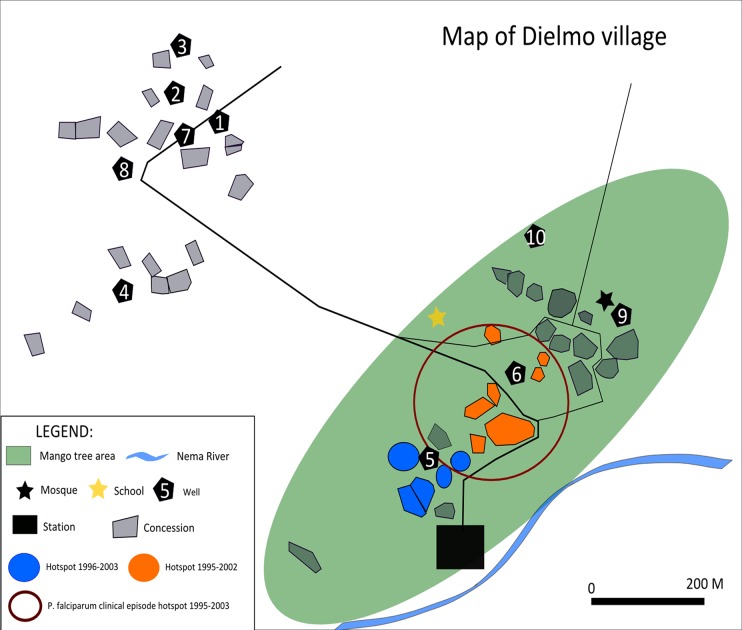
Map of Dielmo village. The village is located at 13°45’N, 16°25’W and is composed of two hamlets showing the houses within the gametocyte hotspots (hotspot 1—blue, 1996–2003, Relative Risk (RR) 1.95, log likelihood ratio (LLR) 14.35 P = 0.0012; cluster 2—green, 1995–2002, RR 1.77, LLR 12.7 P = 0.0053) identified by SaTScan Also shown (large open red circle), area of the *P*. *falciparum* clinical episode hotspot identified by SaTScan; 1995–2003, RR 1.42, LLR 11.01 P = 0.026.

This was performed using Kulldorff's scan statistic in SaTScan (version 9.1.1) (http://www.satscan.org/) [38]. A discrete Poisson model was used to analyze the spatial distribution of gametocyte positive slides amongst all *P*. *falciparum* positive blood slides. The program compares the occurrence of gametocyte positive slides over the number of slides read inside a randomly generated cluster circle compared to the rest of the population. The unit of analysis was the “room” within which the individual lived. Reliable knowledge of room location was available for 308 individuals in 117 rooms spread throughout the village (Fig 1). An infinite number of cluster circles are generated with a maximum diameter set to values between 10m and 250m. A relative risk is calculated and a likelihood ratio test performed. Covariates were not included. A spatio-temporal analysis was performed with temporal time-points set to one year.

## Results

**Fig 2:**
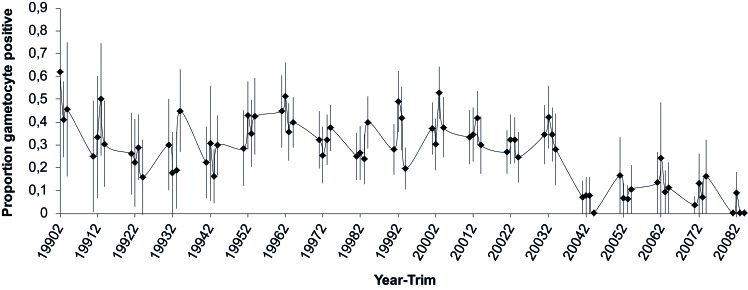
Gametocyte prevalence rate per year-trimester. Shown are means and 95% binomial confidence intervals.

We used the 19-year longitudinal malaria dataset of the Dielmo village cohort, Senegal. From 1990 to 2008, there were 15,567 blood slides read for gametocytes, of which 13,099 were taken during a clinical presentation and 2,468 during a systematic follow-up. This amounted to 2,995 person-trimesters for which parasite positive blood slides were examined for the presence of gametocytes from 544 individuals (median 4 trimester observations per person, range 1–23). The variation in the proportion of blood slides that were positive for gametocytes is shown in Fig 2. 898 person-trimesters included blood slides positive for gametocytes from 297 individuals.

### Global univariate and multivariate analyses

Univariate analyses revealed that gametocyte carriage decreased with age, Fansidar (2004–6) and ACT drug periods (2006–8) and the number of previous infections with *P*. *malariae* and *P*. *ovale*. By contrast, gametocyte carriage increased with the occurrence of a clinical *P*. *falciparum* episode during the trimester, the maximum *P*. *falciparum* and *P*. *ovale* parasite densities during the trimester and ABO blood group (S1 Table). Multivariate analyses using backward elimination of the least informative (least negative AIC) variables one by one, revealed that children (<15 years of age) had significantly higher risk of carrying gametocytes (χ^2^_3_ = 89.9, P<0.001); this was especially so for children ≤5 years of age (OR 3.26; 95% CI 3.00–3.53). The occurrence of a clinical *P*. *falciparum* episode during the trimester (χ^2^_1_ = 46.4, P<0.001) and ABO blood group (A, B or O *vs*. AB) (χ^2^_3_ = 10.9, P = 0.013) increased gametocyte carriage, whereas the Fansidar and ACT drug periods decreased carriage (χ^2^_3_ = 75.4, P<0.001) (Table 1).

**Table 1:** Multivariate Analysis for Risk of Gametocyte Positivity During the Trimester.

**A-** Variable	Category	ORa	95% CI	
Age (years)	< = 5	3.26	3.00	3.53
	]5–10]	2.04	1.75	2.32
	]10–15]	1.72	1.35	2.08
	>15	Ref		
Drug period	Quinine	Ref		
	Chloroquine	1.19	0.99	1.39
	Fansidar	0.35	0.008	0.69
	ACT	0.34	0.005	0.67
*P*. *falciparum* clinical episode in trimester	No	Ref		
	Yes	1.84	1.58	2.10
ABO Blood group	AB	Ref		
	A	1.32	1.04	1.60
	B	1.72	1.44	2.01
	O	1.86	1.63	2.08

Abbreviations: ORa, adjusted Odds Ratio; CI, Confidence Interval; Ref, reference level; ACT, Artemisinin Combination Therapy.

### Spatial cluster analyses

We identified two hotspots of gametocyte carriage: the first within a radius of 37m from 1996–2003 (Relative Risk 1.95 Log likelihood ratio = 14.35, P = 0.0012) that encompassed 33 people in 9 rooms and the second within a radius of 82m from 1995–2002 (Relative Risk 1.77 Log likelihood ratio = 12.7, P = 0.0053) encompassing 60 people in 23 rooms (Fig 1). These hotspots contained 15.0% and 18.2% of the total number of gametocyte positive person-trimesters. The years contributing to the gametocyte hotspots correspond to the Chloroquine drug treatment period (1995–2003).

To take into account any bias occurring through the repeated sampling of the same individual, we also analysed the increased risk of gametocyte carriage in individuals within *vs*. outside of the hotspots by fitting a GLMM as described in the *Statistical analyses* section. Both hotspots were confirmed: hotspot 1 (OR 2.20, 95%CI 1.78–2.62, χ^2^_1_ = 32.6, P<0.001); hotspot 2 (OR 1.68, 95%CI 1.34–2.02, χ^2^_1_ = 18.5, P<0.001).

### Comparative analyses of hotspots

We combined the two hotspots to generate individuals within the high gametocyte density hotspot and those outside of the hotspot. Combined together, of the 93 individuals, only 81 had *P*. *falciparum* positive bloodslides. Of the remaining 215 individuals for whom “room” information was available and who were thus outside of the hotspots, 170 had at least one *P*. *falciparum* positive bloodslide during the same time period. The characteristics, with respect to the identified risk factors, of the individuals with *P*. *falciparum* positive bloodslides within these hotspots compared to those outside within the time period can be seen in Table 2. Notably *P*. *falciparum* positive bloodslides occurred in individuals within the cluster who were younger (73% *vs*. 54% of positive bloodslides occurred in children ≤ 10 years old), who had more *P*. *falciparum* clinical episodes (63% *vs*. 49% positive bloodslides were associated with a clinical episode in the same trimester) and had more B and O blood groups (83% in hotspot *vs* 68% outside hotspot). Thus, within the hotspot, there was a higher frequency of *P*. *falciparum* positive slides occurring in any of the three risk factor groups. Indeed, although the risk associated with these factors was maintained in both areas (Table 2), comparing individuals within and outside of the hotspot during the hotspot period (analysed by nesting the risk factors within site) revealed that both age group and occurrence of a clinical episode had a significantly higher impact on gametocyte carriage within the hotspot (Age group: χ^2^_6_ = 71.6, P<0.001; clinical episode: χ^2^_2_ = 33.3, P<0.001); the effect of ABO blood group tended towards but was not significantly different between sites ((χ^2^_4_ = 9.5, P = 0.053).

**Table 2:** Risk factors for gametocyte positivity individuals inside and outside of the hotspot during the time span of hotspot.

	within cluster in time period				outside cluster in time period			
	*P*.*falciparum* +ve	Gametocyte +ve	%	ORa (95%CI)	*P*.*falciparum* +ve	Gametocyte +ve	%	ORa (95%CI)
**age (years)**								
≤5	241	143	59.3	**2.19 (1.82–2.56)**	535	260	48.6	**1.99 (1.73–2.24)**
]5–10]	118	49	41.5	**1.59 (1.14–2.03)**	330	116	35.2	**1.31 (1.02–1.59)**
]10–15]	47	15	31.9	1.47 (0.77–2.17)	181	45	24.9	1.05 (0.66–1.45)
>15	86	16	18.6	Ref	543	76	14.0	Ref
***P*.*falciparum* episode**								
No	171	46	26.9	Ref	812	146	18.0	Ref
Yes	321	177	55.1	**1.63 (1.14–2.03)**	777	351	45.2	**1.69 (1.47–1.90)**
**ABO group**								
AB & A	83	26	31.3	Ref	481	134	27.9	Ref
B	130	70	53.8	**1.83 (1.38–2.28)**	346	90	26.0	0.99 (0.65–1.32)
O	275	125	45.5	**1.38 (1.06–1.71)**	657	230	35.0	**1.28 (1.05–1.51)**

Shown are the number of person-trimesters for which a blood smear was read for gametocytes and the Odds Ratios from the multivariate analyses.

### Effect of clinical *P*. *falciparum* episodes and time since drug treatment on gametocyte positivity

To assess whether the gametocyte hotspots were directly related to clustering of clinical *P*. *falciparum* episodes, a cluster analysis of the number of *P*. *falciparum* clinical episodes was performed in SaTScan. A hotspot was observed with a radius of 82m from 1995–2003 (RR 1.42 Log likelihood ratio = 11.01, *P* = 0.026) that entirely overlapped the second gametocyte hotspot, encompassing 28 rooms and 73 people (Fig 1).

The overlap of gametocyte positivity during the trimester and the occurrence of a clinical episode during the trimester prompted us to analyse their relationship. Thus, we analysed the entire unaggregated dataset, containing 15,567 *P*. *falciparum* positive bloodslides for which gametocyte positivity had been read. As described previously, the bloodslides comprised both those taken at clinical presentation and during the systematic follow-up. We thus first assessed whether bloodslides taken at clinical presentation *vs*. the systematic follow-up varied in their gametocyte positivity. Taking into account age, ABO blood group and drug period, we fitted a GLMM logistic regression; there was no difference in gametocyte positivity between clinical presentation and systematic follow-up in *P*. *falciparum* positive bloodslides (χ^2^_1_ = 3.4, P = 0.07).

**Fig 3:**
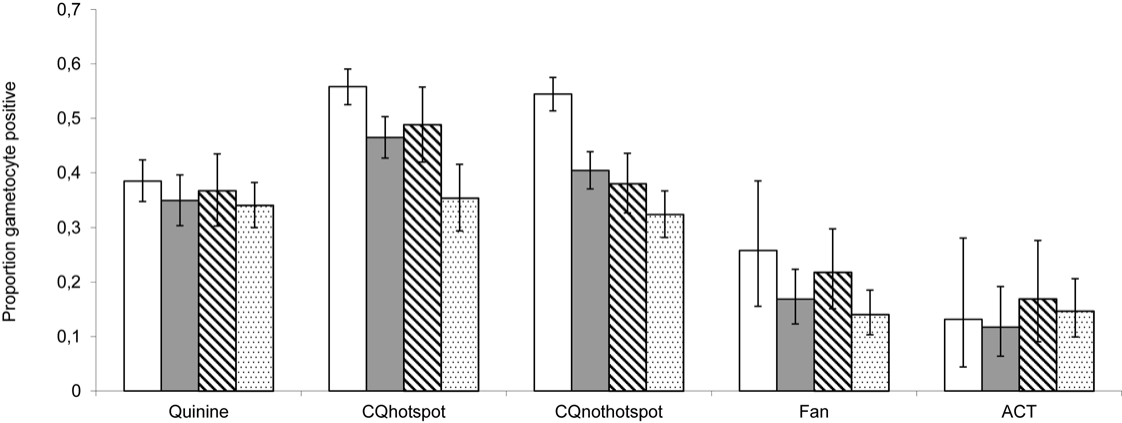
The proportion of *P*. *falciparum* positive bloodslides that are gametocyte positive. This is according to time since last antimalarial drug treatment for each of the drug treatment regimens and separating the Chloroquine drug period into hotspot vs. non hotspot. Also shown are 95% binomial confidence intervals. Open: ≤ 30 days; Grey 30–60 days; Diagonal 61–90 days; Speckled 91–360 days.

Twenty-six percent of parasite positive blood slides had been taken within 30 days of a treated clinical episode. To assess whether the gametocyte hotspot was a result of prior drug treatment, we compared the proportion of bloodslides that were gametocyte positive according to time since last treatment. As can be seen in Fig 3, gametocyte positivity was higher within 30 days of treatment during the Chloroquine drug period as compared to later time points (Previous treatment within 30 days *vs*. treatment >30days ago: In hotspot χ^2^_1_ = 26.2, P<0.001: outside hotspot χ^2^_1_ = 74.2, P<0.001). This was not the case for the other treatment regimens, when there was no difference between proportional gametocyte positivity within 30 days since treatment and at later time points (Quinine χ^2^_1_ = 2.39, P = 0.122; Fansidar χ^2^_1_ = 1.56, P = 0.338; ACT χ^2^_1_ = 0.06, P = 0.827).

We excluded blood slides taken within 30 days since last treatment and tested for the effect of the risk factors identified in the original multivariate analysis. The same gametocyte risk was found in all the previously identified risk factors (Age group: χ^2^_3_ = 96.2, P<0.001; Drug Period χ^2^_3_ = 190.4, P<0.001; ABO blood group χ^2^_3_ = 16.6, P<0.001; clinical case of *P*. *falciparum* within the previous 90 days but excluding that within the previous 30 days χ^2^_1_ = 6.71, P = 0.01).

## Discussion

This study aimed to identify risk factors for *P*. *falciparum* gametocyte carriage from a 19-year long cohort and thus generate potential prognostic key factors that could enable focused intervention. The first key point is that there were distinct hotspots of gametocyte carriage that were spatially and temporally delimited. These hotspots were associated with a hotspot of clinical *P*. *falciparum* episodes, indicating a localised cluster of intense malaria transmission. Secondly, as has been frequently observed, young children had increased risk of gametocyte carriage [15], independently of their increased tendency to have clinical episodes. Thirdly, ABO blood group was significantly associated with gametocyte carriage: blood groups O and B were associated with higher risk of gametocyte carriage than groups A and AB. Finally, gametocyte carriage was significantly increased within 30 days post-treatment with chloroquine.

Many studies have addressed the risk of gametocyte carriage in the weeks following drug treatment; the presence of gametocytes at clinical presentation and treatment failure are recognised risk factors for subsequent gametocyte carriage [39–41]. This seems to be particularly the case following treatment with chloroquine; treatment of chloroquine resistant parasites is associated with a marked increase in gametocyte carriage 7–14 days post-treatment [42–44]. Furthermore, mosquito transmission studies have shown that these resistant post-treatment infections are significantly more infectious to mosquitoes than chloroquine sensitive infections [44, 45]. Laboratory mouse models expressing mutant PfCRT, the parasite gene associated with resistance to chloroquine, have enhanced infectivity to mosquitoes relative to wild type PfCRT in the presence of chloroquine, likely because of protection of immature gametocytes from the lethal action of the drug [46]. Here we do observe a significantly higher gametocyte carriage up to 30 days following chloroquine treatment as compared to gametocyte carriage at clinical presentation at later times since previous treatment, in concordance with previous observations from other study sites [44]. This pattern was not observed for the other drug treatment regimens.

The spatio-temporal analysis revealed gametocyte hotspots that occurred during the chloroquine drug treatment period in a sub-population characterised by an increased prevalence of all the identified risk factors: they were younger, had more clinical *P*. *falciparum* episodes and had a higher proportion of risk ABO blood groups. In light of the known spread of chloroquine-resistant parasites during this period [47], it seems likely that the hotspots were generated by a vicious circle of reduced treatment efficacy of clinical episodes and concomitant increase in post-treatment gametocyte carriage and infectiousness resulting in a high local force of infection. This may have been further exacerbated by the effects of ABO blood group. Although the proximity of the hotspots to the small stream may have contributed to increased exposure to mosquitoes, a previous analysis found no effect of proximity to the stream on density of anophelines [48].

Human genetics and notably sickle cell trait have been previously shown to impact upon gametocyte carriage in this population, but only for asymptomatic infections [24]. Here we found that B and O ABO blood groups were associated with increased gametocyte carriage. This is in line with previous observations of increased gametocyte prevalence in individuals with protective variants (HbS and HbC) of the beta-globin locus [24, 26]. Polymorphisms leading to protection against severe disease seem to be associated with increased propensity to generate gametocytes and hence onwardly transmit the parasite. Blood group O has been associated with protection from severe malaria [25, 49, 50]. It is thought that severe malaria can arise from microvascular obstruction caused by the rosetting of red blood cells induced by the parasite [51]. Rosetting is reduced in individuals with blood group O, intermediate in blood group B, and highest in groups A and AB [52]. The mechanism underlying this increased gametocytogenesis is apparently independent of an effect on asexual parasite density, which was not found to differ according to blood group (Group A: mean 213/μL SEM 92.4; group AB 168 SEM 81.4; group B 177 SEM 77.2; group O: mean 192 SEM 81.4. Values calculated in GLMM after taking into account the other significant variables in the multivariate analysis). Study of the impact of ABO blood group on gametocytogenesis *in vitro* would be informative for understanding the triggers for gametocyte production.

In our study we identified gametocyte carriage in bloodslides that amount to assessing gametocytes in 0.5uL of blood. Molecular techniques have revealed extensive occurrence of sub-microscopic gametocytes [35, 53] that can infect mosquitoes [54] and play an important role as a reservoir of infection especially in areas of seasonal transmission [55]. However, the infectiousness of individuals increases with gametocyte density, especially in asymptomatic infections [56, 57]. Thus, here, whilst we do not capture the entire infectious reservoir, gametocyte positivity, as measured through blood slides, does provide an estimate of the major infectious reservoir.

Identifying hotspots of malaria transmission for targeted intervention offers the potential for drastic reduction in the malaria burden both within and adjacent to the hotspot. The high geographical concentration of individuals with associated risk factors in our study emphasises the importance of addressing fine scale heterogeneity in malaria epidemiology. Such very localised potential transmission hotspots will likely be more resistant to community-based control efforts and require specific targeted action for parasite eradication based on active surveillance of infections as has been recently discussed [58]. This may be no mean feat given the effort required, continuing issues of diagnostics and the high infectiousness of *P*. *falciparum*. In conclusion, whilst rapid access to treatment with an effective anti-malarial can reduce the duration of gametocyte carriage and onward parasite transmission, localised hotspots represent a challenge to malaria control and eventual eradication.

## Supporting Information

S1 Table Univariate analyses for risk of gametocyte positivity during the trimester. *—category; † - Continuous; ‡ - Dichotomous category; ¶ Quartiles.
(DOCX)

S2 Table Multifactorial model simplification method. Shown are the Akaike Information Criterion (AIC) of the model following removal of the variable leading to the largest decrease in AIC at each step (i.e. full model with all 13 variables, then the variable leading to largest decrease in AIC for the 12 variable model and so forth until further removal of variables leads to no further decrease in AIC. The most negative value corresponding to the final model is shown in bold.
(DOCX)
