# Supplementary material for: Risk Factors for Plasmodium falciparum Gametocyte Positivity in a Longitudinal Cohort
Source: PLoS One. 2015 Apr 1;10(4):e0123102. doi: 10.1371/journal.pone.0123102 (PMC4382284; doi:10.1371/journal.pone.0123102)
Supplement: S1 Table — *—category; † - Continuous; ‡ - Dichotomous category; ¶ Quartiles. (DOCX) [file pone.0123102.s001.docx]

**Table S1** Univariate analyses for risk of gametocyte positivity during the trimester. * - category; † - Continuous; ‡ - Dichotomous category; ¶ Quartiles.

| Variable | Univariate GLMM p-value |
| --- | --- |
| Drug period * | <0.0001 |
| Ethnicity * | 0.28 |
| Age * | <0.0001 |
| Gender* | 0.73 |
| Beta-globin status * | 0.96 |
| ABO blood group* | 0.005 |
| Alpha-globin 3.7 deletion* | 0.141 |
| Occurrence of clinical *P. falciparum* episodes in trimester ‡ | <0.0001 |
| Number of *P. falciparum* episodes prior to trimester ¶ | <0.0001 |
| Number of *P. malariae* infections prior to trimester * | <0.0001 |
| Number of *P. ovale* infections prior to trimester * | 0.049 |
| Maximum *P. falciparum* parasite density in trimester † | <0.0001 |
| Maximum *P. malariae* parasite density in trimester † | 0.09 |
| Maximum *P. ovale* parasite density in trimester † | 0.78 |
| Maximum *P. malariae* gametocyte density in trimester † | 0.15 |
| Maximum *P. ovale* gametocyte density in trimester † | 0.32 |
| Number of days since last *P. ovale* treatment † | <0.0001 |
| Number of days since last *P. malariae* treatment † | <0.0001 |
| Number of days of follow up in trimester † | 0.65 |
