# Supplementary material for: Risk Factors for Plasmodium falciparum Gametocyte Positivity in a Longitudinal Cohort
Source: PLoS One. 2015 Apr 1;10(4):e0123102. doi: 10.1371/journal.pone.0123102 (PMC4382284; doi:10.1371/journal.pone.0123102)
Supplement: S2 Table — Shown are the Akaike Information Criterion (AIC) of the model following removal of the variable leading to the largest decrease in AIC at each step (i.e. full model with all 13 variables, then the variable leading to largest decrease in AIC for the 12 variable model and so forth until further removal of variables leads to no further decrease in AIC. The most negative value corresponding to the final model is shown in bold. (DOCX) [file pone.0123102.s002.docx]

**Table S2** Multifactorial model simplification method (GLMM). Shown are the Akaike Information Criterion (AIC) of the model following removal of the variable leading to the largest decrease in AIC at each step (i.e. full model with all 13 variables, then the variable leading to largest decrease in AIC for the 12 variable model and so forth until further removal of variables leads to no further decrease in AIC. The most negative value corresponding to the final model is shown in bold.

| Number of variables in model | Variable removed | AIC |
| --- | --- | --- |
| 13 | Full model | -472 |
| 12 | Number of days since last *P. ovale* treatment | -850 |
| 11 | Number of days since last *P. malariae* treatment | -1214 |
| 10 | Alpha-globin 3.7 deletion | -1809 |
| 9 | Number of *P. ovale* infections prior to trimester | -1861 |
| 8 | Maximum *P. falciparum* parasite density in trimester | -1882 |
| 7 | Number of *P. falciparum* episodes prior to trimester | -1902 |
| 6 | Number of *P. malariae* infections prior to trimester | -1912 |
| 5 | Maximum *P. malariae* parasite density in trimester | -1931 |
| 4 | Maximum *P. malariae* gametocyte density in trimester | **-1941** |
